# Cancer Immunotherapy and Identification of Prognostic and Predictive Biomarkers

**DOI:** 10.1155/2018/5184075

**Published:** 2018-03-20

**Authors:** Carmen Criscitiello, Michele Santangelo, Fotios Loupakis

**Affiliations:** ^1^Division of Early Drug Development, European Institute of Oncology, Milano, Italy; ^2^Department of Advanced Biomedical Science, University of Naples Federico II, Via S. Pansini 5, 80131 Naples, Italy; ^3^Medical Oncology Unit 1, Clinical and Experimental Oncology Department, Veneto Institute of Oncology IOV-IRCCS, Padua, Italy

The immune system is naturally able to detect and destroy abnormal cells thus preventing the development of many cancers. Nevertheless, sometimes cancer cells may avoid detection and destruction by the immune system. Cancer cells may decrease the expression of tumor antigens on their surface, making it harder for the immune system to detect them, express on their surface proteins that induce immune cell inactivation, induce cells in the microenvironment to release factors that suppress immune responses, and promote tumor cell proliferation and survival. In the past few years, the field of cancer immunology has rapidly advanced and led to several new treatment strategies for cancer patients, which may increase the strength of immune responses against tumors. Immunotherapies may work by either stimulating the activities of specific components of the immune system or by counteracting signals produced by cancer cells that suppress immune responses. These advances in cancer immunotherapy have been made possible thanks to huge long-term investments in basic research on the immune system. But, the research in the immunotherapy field is still extremely active. There are still many open questions requiring additional intense research to understand why immunotherapy is effective only in a minority of cancer patients. Prognostic and predictive biomarkers are needed to identify those patients who will derive the greatest benefit from an immunotherapy-based approach. In addition, ongoing research is focusing on expanding the use of immunotherapy to more cancer types. Another crucial area of research aims at increasing the effectiveness of immunotherapy by combining it with other anticancer treatments, such as targeted therapy, chemotherapy, and radiation therapy as well as combinations of different types of immunotherapy agents. In the last year, the US Food and Drug Administration (FDA) approved the first adoptive cell immunotherapy, the so-called chimeric antigen receptor (CAR) T-cell therapy, and granted its first tissue/site-agnostic approval (i.e., a treatment working against different types of cancers that share a common genetic abnormality) for a drug [1]. Indeed, pembrolizumab was approved for use in adult and pediatric patients with locally advanced or metastatic solid tumors that are mismatch-repair deficient or microsatellite instability-high (MSI-H) who have progressed after prior treatment and who have no satisfactory alternative treatment options [2]. This represents a paradigm shift in cancer drug approvals, highlighting the concept that a biomarker may define the disease better than the site.

The first paper of this special issue reports current evidence and future perspectives for advanced urothelial cancer patients treated with immunotherapy. The second paper analyzes current tissue molecular markers in colorectal cancer. The third paper highlights the importance of mismatch repair deficiency as a predictive biomarker for immunotherapy efficacy. Gastric cancer is the topic of the fourth and fifth articles of this special issue: the fourth one reviews preclinical and clinical recent development of immunotherapeutic strategies in gastric carcinoma and the fifth one summarizes which are the diagnostic, predictive, prognostic, and therapeutic molecular biomarkers in third millennium for this disease. The sixth published paper studies heterogeneous periostin expression in different histological variants of papillary thyroid carcinoma. The seventh paper tries to foresee which might be the clinical applications of immunotherapy combination in the future. The eighth paper reviews evidence on the role of immunotherapy in gastrointestinal cancers.

This special issue wants to highlight how immunotherapy research spans the continuum from basic scientific research to clinical research applications. It is paramount to keep on working to foster the discovery, development, and delivery of immunotherapy approaches to treat cancer.

*Carmen Criscitiello* *Michele Santangelo* *Fotios Loupakis*
